# Analgesic and Anti-Inflammatory Activities of Quercetin-3-methoxy-4′-glucosyl-7-glucoside Isolated from Indian Medicinal Plant *Melothria heterophylla*

**DOI:** 10.3390/medicines6020059

**Published:** 2019-05-27

**Authors:** Arijit Mondal, Tapan Kumar Maity, Anupam Bishayee

**Affiliations:** 1 Department of Pharmacy, NSHM Knowledge Campus, Kolkata-Group of Institutions, Kolkata 700053, India; 2 Department of Pharmaceutical Technology, Jadavpur University, Kolkata 700032, India; jutkmaity@yahoo.com; 3 Lake Erie College of Osteopathic Medicine, Bradenton, FL 34211, USA

**Keywords:** *Melothria heterophylla*, quercetin-3-methoxy-4′-glucosyl-7-glucoglucoside, analgesic, anti-inflammatory, cyclooxygenase, rats, mice

## Abstract

**Background:***Melothria heterophylla* (family: Cucurbitaceae), commonly known as kudari, is used in the Indian traditional medicine to treat various inflammation-associated diseases, such as asthma, arthritis and pain. However, the anti-inflammatory active components of this plant have not been identified yet. The aim of this study was to investigate the potential analgesic and anti-inflammatory activities of a compound, quercetin-3-methoxy-4′-glucosyl-7-glucoside, isolated from *M. heterophylla*. **Methods:** The anti-inflammatory activity was determined using carrageenan- and dextran-induced rat paw edema as well as cotton pellet-induced granuloma in rats, whereas the analgesic activity was analyzed using acetic acid-induced writhing, hot plate and tail flick response in mice. The test compound was orally administered at a dose of 5, 10 or 15 mg/kg. The cyclooxygenase-1 (COX-1)- and COX-2-inhibitory capacity of the test compound was studied by enzyme immunosorbent assay. **Results:** Quercetin-3-methoxy-4′-glucosyl-7-glucoglucoside at 15 mg/kg exhibited a maximum inhibition of carrageenan-induced inflammation (50.3%, *p* < 0.05), dextran (52.8%, *p* < 0.05), and cotton pellets (41.4%, *p* < 0.05) compared to control animals. At the same dose, it showed a 73.1% inhibition (*p* < 0.05) of the pain threshold in acetic acid-induced writhing model. It also exhibited a considerable analgesic activity by prolonging the reaction time of the animals based on hot plate as well as tail flick response. The test compound was found to inhibit COX-1 (IC_50_ 2.76 µg/mL) and more efficiently, COX-2 (IC_50_ 1.99 µg/mL). **Conclusions:** Quercetin-3-methoxy-4′-glucosyl-7-glucoside possessed substantial analgesic and anti-inflammatory activities possibly due to inhibition of prostaglandin production, supporting the ethnomedicinal application of *M. heterophylla* to treat various inflammatory disorders.

## 1. Introduction

Inflammation represents a complex biological protective response of the body to harmful stimuli introduced to the host. These noxious stimulants include radiation, chemical, physical, infectious and immunological incitation. Inflammatory ailments, such as allergy, asthma, hepatitis, autoimmune diseases, inflammatory bowel disease, coeliac disease, glomerulonephritis, preperfusion injury, transplant rejection and rheumatic disorders, affect a significant portion of the global population [1]. Chronic inflammation leads to various metabolic ailments, including obesity, cardiovascular, neurodegenerative diseases and cancer [2,3]. The inducers of inflammation initiate the inflammation process by stimulating inflammatory cells to produce elevated levels of proinflammatory cytokines, including interleukin-1β (IL-1β), IL-6 and tumor necrosis factor-α (TNFα), whereas inflammatory mediators, such as nitric oxide (NO), inducible nitric oxide synthase (iNOS), cyclooxygenase-1 (COX-1) and COX-2, affect the functionality of tissues and organs [4].

The non-steroidal anti-inflammatory drugs (NSAIDs) are commonly used for their analgesic, anti-inflammatory and antipyretic activities. Many NSAIDs pose a significant risk of toxicity following acute and chronic use. Medicinal plants contain an assortment of compounds with promising biological and pharmacological activities [5]. Herbal medicines are advantageous because of their low cost and fewer adverse effects [6,7]. Numerous medicinal plants and their phytoconstituents are endowed with potent anti-inflammatory activities useful for treating various inflammatory diseases [8,9]. Traditional medicines play an important role in treating inflammation-linked diseases [10]. Therefore, present day investigation with indigenous plant metabolites can open up new frontiers in the treatment of inflammation, which can be beneficial in the management of rheumatism, arthritis and pain.

*Melothria heterophylla* (Lour.) Cogn. (a plant of Cucurbitaceae family), generally known as kudari, is a scandent herb with tuberous roots which grow in various parts of India. The leaves of this plant find use in ethnomedicine due to stimulating, invigorating and purgative effects [11]. A juice prepared from the leaves of *M. heterophylla* is known to possess anti-inflammatory properties [12]. The ethanolic extract of the aerial parts of *M. heterophylla* has been found to provide hepatoprotection against carbon tetrachloride-induced liver injury in rats [13]. It also exhibits antitumor effects against mouse Ehrlich ascites carcinoma by proapoptotic activity mediated by activation of caspase-3 [14]. It is reported that the plant has profound hypoglycemic activity in streptozotocin-induced diabetic rats [15]. The plant also possesses significant in vitro antioxidant activity [16,17]. The aerial parts of this plant have been found to possess anthelmintic activity [18]. However, the anti-inflammatory active components of this plant have not been identified yet.

In view of the ethnobotanical uses of the plant in the treatment of pain and inflammation, this study was undertaken to evaluate potential analgesic and anti-inflammatory effect of an isolated secondary metabolite using several acute and chronic in vivo models. Additionally, we explored a possible mechanism of action of an isolated compound to assess the justification of the use of this plant as a topical anti-inflammatory agent in traditional medicine.

## 2. Materials and Methods

### 2.1. Plant Material

The young matured plants of *M. heterophylla* were collected from the rural areas of Mayurbhanj district (Odisha, India) during August and September in 2017. The materials were identified by Dr. Potharaju Venu, a taxonomist of Botanical Survey of India (Howrah, India). A voucher specimen [CNH/I-I(65)2006/Tech.II/1661] was deposited at the Department of Pharmaceutical Technology, Jadavpur University (Kolkata, India). All collected plant materials (whole aerial parts including roots) were washed, dried under the shade and subsequently pulverized to course powder by using a mechanical processor.

### 2.2. Extraction and Compound Isolation

The powdered plant material (2 kg) was defatted using petroleum ether (60–80 °C) and subsequently extracted with 4 L of ethanol (95%) in a Soxhlet apparatus. Under reduced pressure, the solvent was removed to acquire petroleum ether (PEMH, yield 3.42%) and ethanolic (EEMH, yield 40.2%) extract. The ethanolic extract was apportioned progressively between an ethyl acetate-water system and then between *n*-butanol-water system (3 × 1 L). The respective solvents were removed under reduced pressure to yield ethyl acetate fraction (EAF, 112 g) and *n*-butanol fraction (NBF, 73.4 g). Both the fractions were assessed for anti-inflammatory activity. NBF was found to be more potent than EAF. The NBF fraction (4 g) was fractionated over the silica gel column and eluted with hexane-ethyl acetate (25:1-1:1) to obtain five different fractions. Fraction 3 (1 g) was subjected to silica gel column chromatography and eluted with hexane-acetone (10:1.5), which led to the isolation of one secondary metabolite as a yellowish amorphous powder. This compound was characterized as quercetin-3-methoxy-4′-glucosyl-7-glucoside (60 mg) based on its melting point and data generated by UV spectroscopy, infrared (IR) spectroscopy, mass spectrometry (MS) and ^1^H-, and ^13^C-nuclear magnetic resonance (NMR).

### 2.3. Animals and Maintenance

Male Wistar albino rats, weighing 150–250 g, and male Swiss albino mice, weighing 18–20 g were utilized for various studies. The animals were housed in polyacrylic cages with not more than six animals/cage. The animals were acclimatized to standard laboratory conditions (temperature 25 ± 2 °C) with dark/light cycle (14/10 h) for one week before the commencement of an experiment. The animals had free access to standard dry pellet diet (Hindustan Lever, Kolkata, India) and water *ad libitum*. All animal experiments were conducted following procedures approved by the Animal Ethical Committee of Jadavpur University (CPCSEA/ORG/CH/2006/Reg.No.95, date of approval: 15 May 2008).

### 2.4. Chemicals

Aspirin (USV Health Care Company, Mumbai, India), morphine (M.M Pharma, Chennai, India) and indomethacin (Shreeji Pharma International, Vadodara, India) were used as the standard drugs. Carrageenan was obtained from S.D. Fine Chemicals Limited (Mumbai, India), whereas histamine and dextran were purchased from Sigma-Aldrich (St. Louis, MO, USA). Various other chemicals and reagents were of analytical grade and acquired from local firms.

### 2.5. Assessment of Analgesic Activities

Assessment of analgesic properties of the test compound were performed utilizing chemical, mechanical and thermal noxious stimuli as described below.

#### 2.5.1. Acetic Acid-Induced Writhing Method

An acetic acid-induced writhing experiment was executed according to the method of Koster et al. [19] with slight modification. Five groups of six mice in each were selected as follows: Group 1 received the vehicle (normal saline, 5 mL/kg, orally), groups 2, 3 and 4 had three doses of the test compound (5, 10, and 15 mg/kg, orally, respectively) and the standard drug acetyl salicylic acid (10 mg/kg, orally) was administered in group 5. Writhing was induced in all animals by intraperitoneal (i.p.) injection of acetic acid solution (0.2 mL of 3% acetic acid) 1 h following normal saline, test compound or drug treatment. After acetic acid administration, the animals were placed in transparent boxes, the number of writhes was recorded for a period of 20 min and the percentage inhibition was determined.

#### 2.5.2. Hot Plate Method

Five groups of six mice each were utilized for this study. Group 1 (control) animals received the vehicle (normal saline, 5 mL/kg, orally), whereas the test compound was administered orally to groups 2, 3 and 4 at a dose of 5, 10 and 15 mg/kg, respectively. Group 5 animals received the standard drug morphine (5 mg/kg, subcutaneously (s.c.)). The animals were positioned on an aluminum hot plate maintained at a temperature of 55 ± 0.5 °C for a maximum time of 30 s. Reaction time was recorded when the animals licked their fore- and hind-paws and bounced out of the hot plate before and 15, 30, 45 and 60 min following administration of test agents [20]. A similar method was followed for group 1 after the administration of normal saline. The animals which responded within 15 s and did not have large variation in reaction time when tested on four separated events were chosen for this study.

#### 2.5.3. Tail Flick Response

Five groups of six mice each were randomly selected for this study. Group 1 received the vehicle (normal saline, 5 mL/kg, orally). The test compound was orally administered to groups 2, 3 and 4 at a dose of 5, 10 and 15 mg/kg, respectively. The standard drug morphine (5 mg/kg, s.c.) was administered to group 5. Analgesic activity was estimated 30 min following the administration of normal saline, test or standard drug [21]. The tail of each mouse was positioned on the nichrome wire of an analgesiometer (Techno, Lucknow, Uttar Pradesh, India), which was set at 5.5 ± 0.5 amp. The time taken by an animal to pull back (flick) its tail from the hot wire was taken as the response time. A reading was taken following 30 min of administration of the normal saline, test compound or standard drug to the respective groups. The animals which responded within 15 s and didn’t demonstrate large variation were chosen for this experiment.

### 2.6. Evaluation of Anti-Inflammatory Activities

Three separate experiments were performed to assess anti-inflammatory activity as presented in the following sections.

#### 2.6.1. Carrageenan-Induced Rat Paw Edema

Six groups of six rats each were administered with the vehicle (normal saline, 5 mL/kg, p.o.), the test compound (5, 10 or 15 mg/kg, p.o.), and indomethacin (10 mg/kg, p.o.). One hour following the treatment with various agents, edema was induced by a subplantar injection of 0.1 mL of 1% freshly prepared suspension of carrageenan into the right hind paw of each animal. The volume of the injected paws was estimated at 0 and 3 h following carrageenan injection utilizing a plethysmometer (Ugo Basile, Gemonio, Varese, Italy) as per the technique of Winter et al. [22]. The extent of edema development was determined by the increase in paw volume. The increase in paw volume as well as percentage inhibition was calculated utilizing the following equations:

Increase in paw volume in control (Pc) = Pt – Po

Increase in paw volume in treated (PT) = Pt – Po

Percentage inhibition = [(Pc – PT)/Pc] × 100

Where, Pt is the paw volume at time t, Po is initial paw volume.

#### 2.6.2. Dextran-Induced Rat Paw Edema

The experimental animals were treated in a way like that of the carrageenan-induced paw edema model where dextran (0.1 mL, 1 % w/v in normal saline) was utilized instead of carrageenan [23].

#### 2.6.3. Cotton Pellet-Induced Granuloma

The cotton pellet-induced granuloma in rats was studied according to the method described by D’Arcy et al. [24]. The rats were divided into five groups of six animals in each. The rats were anesthetized and sterile cotton pellets weighing 10 ± 1 mg were implanted (s.c.) into both sides of the groin area of each rat. Group 1 animals served as a control and received the vehicle (normal saline, 5 mL/kg, p.o.). The test compound at a dose of 5, 10 and 15 mg/kg was administered orally to groups 2, 3, and 4, respectively, for seven successive days from the day of cotton pellet implantation. Group 5 animals were treated with indomethacin at a dose of 10 mg/kg (p.o.) for a similar period. On the eighth day, the rats were anesthetized and the pellets together with the granuloma tissues were carefully removed and made free from extraneous tissues. Subsequently, the wet pellets were weighed and dried in an oven at 60 °C for 24 h to obtain constant weight. The increment in the dry weight of the pellets was considered to be the measure of granuloma development.

### 2.7. COX-1 and COX-2 Inhibitory Assay

The Cayman COX inhibitory assay was performed to study the ability of a test agent to inhibit COX-1 and COX-2 according to the protocol of the Cayman Chemical Company (Ann Arbor, MI, USA) [25]. The assay utilized both ovine COX-1 and human recombinant COX-2 enzymes to screen isozyme-specific inhibitors. Five different concentrations of the test compound (2.5–20 µg/mL) were prepared by dissolving the compound in dimethyl sulfoxide. The colometric estimation was performed at 412 nm to measure the level of prostaglandin F2α (PGF2α), which was produced due to the enzymatic reaction. The percentage inhibition was calculated.

### 2.8. Statistical Analysis

Values are presented as mean ± standard error of mean (SEM). A statistical significance was determined by one-way analysis of variance (ANOVA), followed by a Student’s *t*-test. A *p*-value lower than 0.05 was considered statistically significant.

## 3. Results

### 3.1. Structural Elucidation of the Test Compound

**Figure 1:**
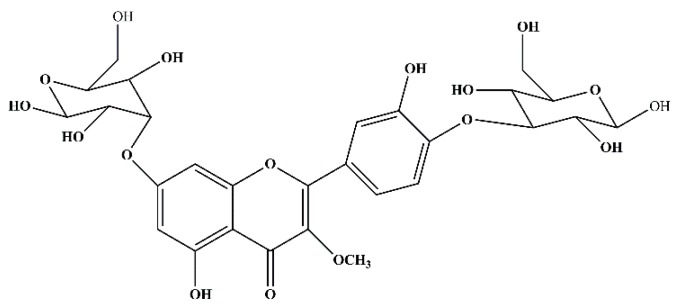
The structure of quercetin-3-methoxy-4′-glucosyl-7-glucoside.

The structure of the test compound was determined by UV, IR, MS, and ^1^H- and ^13^C-NMR analysis as quercetin-3-methoxy-4′-glucosyl-7-glucoside (Figure 1 and Figures S1–S3 in Supplementary Materials). This compound was obtained as a yellow amorphous powder. UV λ_max_238 nm (CH_3_OH): m.p: 159–163 °C. IR (KBr) v cm^−1^ 3412.78 (-OH), 2952.51 (-OCH_3_, C-H stretch), 1680.15 (ketone C=O stretch), 1605.36 (C=C-C, aromatic ring stretch), 1102.74 (alkyl substituted ether, C-O stretch), 799.24 (aromatic C-H out of plane bends), 466.61 (out of plain ring bending). ^1^H-NMR (DMSO-d6): δ_H_ 7.21–6.88 (2H, m, H15, H14), 6.74 (1H, s, H6), 6.29 (1H, s, H2), 5.41 (1H, s, H23), 5.35 (1H, s, H18), 3.96–3.91 (4H, m, H21, H22, H35, H36), 3.82–3.76 (2H, d, J = 7Hz, H37, H38), 3.80 (3H, s, OCH_3_11), 3.65–3.58 (2H, m, H28, H26), 3.61 (2H, s, H41), 3.58 (1H, s, H39). ^13^C- NMR (DMSO-d6): δ_C_ 178.2 (C7), 165.3 (C1), 161.0 (C3), 158.0 (C5), 154.9 (C9), 149.0 (C16), 147.2 (C17), 139.6 (C8), 121.8 (C12), 121.0 (C14), 114.5 (C18), 112.2 (C15), 103.5 (C4), 98.8 (d, C24, C35), 98.1 (C2), 92.9 (C6), 81.5 (d, C26, C39), 78.3 (C22), 78.0 (C37), 73.4 (C23), 68.9 (d, C21, C38), 62.2 (C41), 58.6 (OCH_3_11) ppm. ESI-HRMS (m/z) (M + H)^+^: Found: 641.17186. Calc. for C_28_H_33_O_17_: 641.16.

### 3.2. Analgesic Activity

The test compound at a dose of 5, 10 or 15 mg/kg showed significant and dose-dependent antinociceptive activity in all the three separate models for nociception. Table 1 indicates that the test compound significantly decreased abdominal constrictions initiated by the i.p. administration of acetic acid. The observed effect of the test compound was dose-dependent and at maximum dose (15 mg/kg) it produced a practically comparable effect to the standard drug acetyl salicylic acid.

As presented in Table 2, treatment of animals with the test compound prolonged the animal’s reaction time to the heat stimulus in a dose-responsive fashion. The highest reaction time was noticed for the test compound at 60 min after the administration of each dose. At this time point, the result with the test compound at 15 mg/kg was observed to be similar to that of the standard drug morphine.

The effects of the test compound on the tail flick response of the mice is depicted in Table 3. The results exhibited that the administration of the test compound at various doses (5, 10 or 15 mg/kg) significantly increased the mouse tail response time when the animal’s tail was exposed to heat generated by the tail flick device. The test compound at a dose of 15 mg/kg produced a comparative effect to that of the standard drug morphine.

### 3.3. Anti-Inflammatory Activity

**Figure 2:**
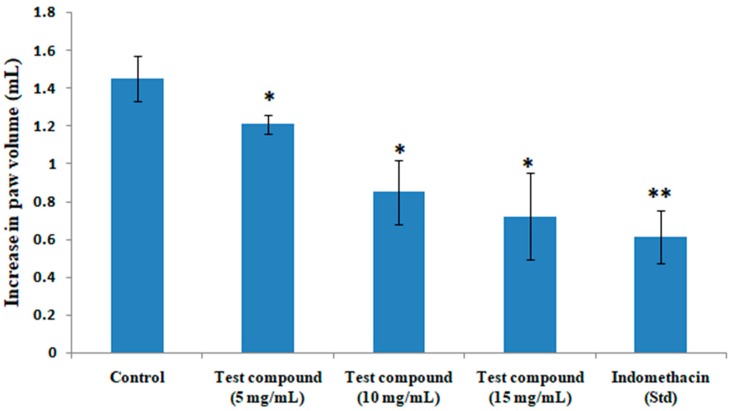
Effects of quercetin-3-methoxy-4′-glucosyl-7-glucoside on carrageenan-induced paw edema in rats. The results represent mean ± SEM (n = 6). * *p* < 0.05 and ** *p* < 0.01 compared to the control group.

The anti-inflammatory activities of the test compound at a dose of 5, 10 or 15 mg/kg against acute paw edema in rats initiated by carrageenan is depicted in Figure 2. The isolated compound produced a noteworthy and dose-dependent decrease in rat paw edema compared to the control. The anti-inflammatory effect exhibited by the test compound at a dose of 15 mg/kg was found to be similar to that of the standard drug indomethacin.

**Figure 3:**
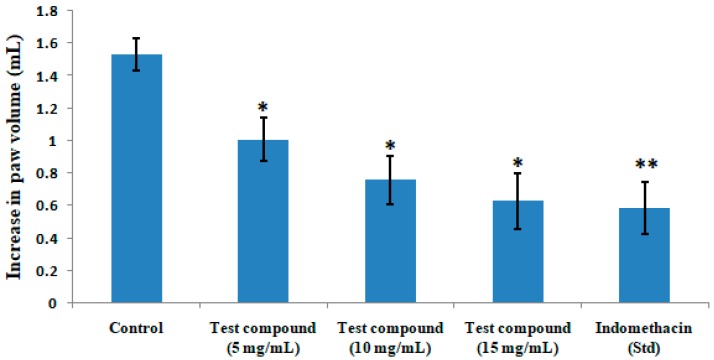
Effects of quercetin-3-methoxy-4′-glucosyl-7-glucoside on dextran-induced paw edema in rats. The results represent mean ± SEM (n = 6). * *p* < 0.05 and ** *p* < 0.01 compared to the control group.

The results on paw volume following the administration of the test compound or standard drug indomethacin in dextran-induced rat paw edema model is summarized in Figure 3. The test compound produced a significant and dose-dependent decrease in paw volume in rats. The test compound at a dose of 15 mg/kg showed a similar effect to that of the standard drug indomethacin.

A significant decrease in the weight of cotton pellets was noticed with the test compound in comparison to the vehicle-treated rats (Table 4). The test compound showed a similar effect in terms of the decrease in the weight of cotton pellets at a dose of 15 mg/kg to that of the standard drug indomethacin.

**Figure 4:**
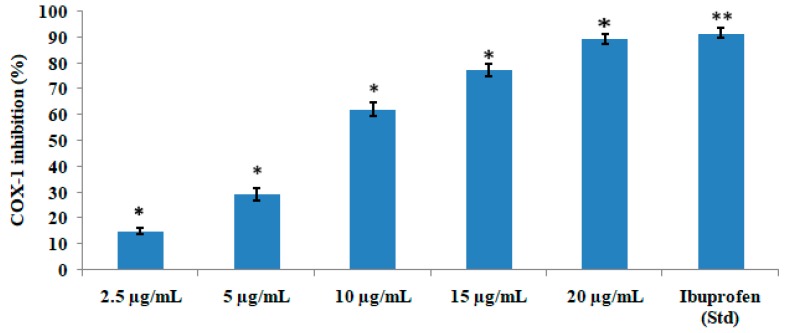
COX-1-inhibitory activities of various concentrations of quercetin-3-methoxy-4′-glucosyl-7-glucoside. The results represent mean ± SEM (n = 3). * *p* < 0.05 and ** *p* < 0.01 compared to the control group.

**Figure 5:**
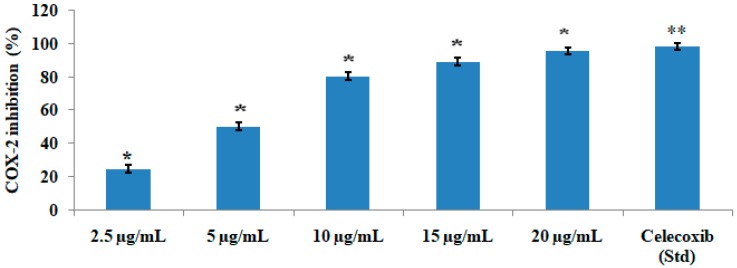
COX-2-inhibitoty activities of various concentrations of quercetin-3-methoxy-4′-glucosyl-7-glucoglucoside. The results represent mean ± SEM (n = 3). * *p* < 0.05 and ** *p* < 0.01 compared to the control group.

The test compound significantly (*p* < 0.05) and concentration-dependently inhibited the COX-1 (Figure 4) and COX-2 (Figure 5) enzyme. The IC_50_ value was found to be 2.76 µg/mL and 1.99 µg/mL for COX-1 and COX-2, respectively, which showed that the compound inhibited COX-2 more effectively than COX-1. The test compound at a concentration of 15 µg/mL showed 77.25% inhibition of COX-1 in comparison with the standard drug ibuprofen, which showed 91.56% enzyme inhibition. The same test compound at the same concentration of 15 µg/mL exhibited 89.34% inhibition of COX-2 in comparison with 98.31% inhibition by the standard drug celecoxib.

## 4. Discussion

Bioactive plant extracts represent valuable resources for the development of anti-inflammatory agents, which could be helpful in the management of pain and inflammation. *M. heterophylla* (Lour.) Cogn., belonging to the Cucurbitaceae family, is a scandent dioecious perennial herb with several tuberous roots and slender branched furrowed stems bearing simple tendrils. According to the ethnomedicinal database, the leaves of *M. heterophylla* were reported to possess analgesic and anti-inflammatory activities [12]. Gallic acid, rutin and β-sitosterol were previously isolated from this plant [14]. Additionally, another group [16] reported the presence of 1,2,4,6-tetra-O-galloyl-β-(D)-glucopyranose in the ethyl acetate soluble fraction of the ethanolic extracts of *M. heterophylla*. The active constituents responsible for analgesic and anti-inflammatory activity have not been recognized yet. Hence, we initiated the present work to isolate one active phytoconstituent of *M. heterophylla* and probe into a possible analgesic and anti-inflammatory effects as well as underlying mechanisms of action. Our bioassay-guided isolation approach led to the separation of quercetin-3-methoxy-4′-glucosyl-7-glucoside, which was evaluated for its analgesic and anti-inflammatory activities and associated mechanism of action.

The writhing initiated by synthetic substances is because of sensitization of nociceptors by prostaglandins. Various inflammatory mediators, such as prostaglandin, proinflammatory cytokines and chemokines, are known to instigate pain through direct activation of nociceptors, the essential sensory neurons that detect noxious stimuli [26]. In this study, the test compound exhibited substantial inhibitory activity on the writhing response prompted by acetic acid when compared to that of the control. The observed analgesic effect of the test compound could be due to its anti-inflammatory action similar to the effect of salicylates, which are especially effective in relieving the type of pain linked to inflammation or edema [16]. Furthermore, the test compound registered significant analgesic activity in comparison with the standard drug based on hot plate and tail flick studies. It is well-known that centrally-acting antinociceptive drugs increase the pain threshold of animals towards heat and pressure [27].

Carrageenan and dextran-induced edema models were utilized to determine the anti-inflammatory activity of the test compound in vivo. It is well-established that the development of edema inflicted by carrageenan is a three-stage process: The early stage (the first 90 min) during which histamine and serotonin are released; the second stage (90–150 min) which is driven by kinin; and the third stage (after 180 min), which is mediated by prostaglandin [28,29]. Our results indicated that the test compound was likely to act by hindering the release and/or action of prostaglandin.

The development of granuloma in rodents by cotton pellet represents a chronic inflammation model extensively used to assess the transudative and proliferative components of the inflammation. The weight of the cotton pellets corroborates with the amount of the granulomatous tissue [28]. Based on this study, administration of the test compound was effective in lowering the weight of the cotton pellet. These data have suggested the anti-inflammatory effect of the test compound.

Activation of COX enzymes increases the level of prostaglandin F2α (PGF2α), also known as amoglandin, which causes inflammation and pain. The ovine/human COX-inhibitory assay is a direct estimation of PGF2α production from prostaglandin H2 (PGH2). The prostanoid product was evaluated by enzyme immunosorbent assay (ELISA) utilizing a broadly specific antibody that binds to all the major prostaglandin compounds. It primarily incorporates both ovine COX-1 and human recombinant COX-2 enzymes to screen isozyme-specific inhibitors. The test compound quercetin-3-methoxy-4′-glucosyl-7-glucoside showed significant anti-inflammatory activity since it inhibited COX-1 and COX-2. However, the compound inhibited COX-2 at a much lower concentration, indicating that it is more specific towards the COX-2 pathway.

## 5. Conclusions

This study demonstrated that quercetin-3-methoxy-4′-glucosyl-7-glucoside isolated from *M. heterophylla* exhibited analgesic activity against nociceptive responses triggered in mice by chemical (acetic acid injection), mechanical and thermal noxious stimuli. It also showed anti-inflammatory activity against carrageenan-induced paw edema, dextran-induced rat paw edema and cotton pellet-induced granuloma. It reduced the amount of arachidonic acid transformed to PGs by suppressing COX-2 level more effectively in comparison to COX-1. The observed analgesic and anti-inflammatory activities of quercetin-3-methoxy-4′-glucosyl-7-glucoside has been found to be comparable to those of standard drugs. Thus, *M. heterophylla* possesses significant analgesic and anti-inflammatory activities, which supports the use of this plant in ethnomedicine for the treatment of various inflammation-driven disorders.

## Figures and Tables

**Table 1:** Effect of quercetin-3-methoxy-4′-glucosyl-7-glucoside on acetic acid-induced writhing in mice.

Treatment	Number of Writhes	Inhibition (%)
Control	45.12 ± 0.02	-
Test Compound (5 mg/kg)	28.13 ± 0.31 *	38
Test Compound (10 mg/kg)	20.71 ± 0.32 *	54
Test Compound (15 mg/kg)	12.14 ± 0.01 *	73
Acetyl Salicylic Acid (10 mg/kg)	10.23 ± 0.02 **	77

The results are represented by mean ± SEM (n = 6). * *p* < 0.05 and ** *p* < 0.01 compared to control group.

**Table 2:** Antinociceptive activity of quercetin-3-methoxy-4′-glucosyl-7-glucoside analyzed by hot plate method in mice.

Treatment	Latency Period (sec)				
	At 0 min	After 15 min	After 30 min	After 45 min	After 60 min
Control	6.40 ± 0.1	6.49 ± 0.5	6.12 ± 0.3	5.89 ± 0.5	5.73 ± 0.4
Test Compound (5 mg/kg)	6.42 ± 0.12 *	7.75 ± 0.2 *	8.39 ± 0.11 *	8.99 ± 0.23 *	9.12 ± 0.31 *
Test Compound (10 mg/kg)	6.38 ± 0.21 *	8.01 ± 0.17 *	8.89 ± 0.12 *	9.33 ± 0.2 *	10.33 ± 0.33 *
Test Compound (15 mg/kg)	6.43 ± 0.14 *	9.57 ± 0.5 *	10.27 ± 0.3 *	11.52 ± 0.12 *	12.56 ± 0.23 *
Morphine (5 mg/kg)	6.41 ± 0.32 **	11.45 ± 0.3 **	13.02 ± 0.21 **	13.29 ± 0.1 **	13.45 ± 0.1 **

The results are represented by mean ± SEM (n = 6). * *p* < 0.05 and ** *p* < 0.01 compared to control group.

**Table 3:** Effect of quercetin-3-methoxy-4′-glucosyl-7-glucoside on tail flick response in mice.

Treatment	Reaction Time (sec)
Control	4.3 ± 0.12
Test Compound (5 mg/kg)	5.9 ± 0.3 *
Test Compound (10 mg/kg)	7.5 ± 0.11 *
Test Compound (15 mg/kg)	8.3 ± 0.23 *
Morphine (5 mg/kg)	8.31 ± 0.3 *

The results are represented by mean ± SEM (*n* = 6). * *p* < 0.05 compared to control group.

**Table 4:** Effect of quercetin-3-methoxy-4′-glucosyl-7-glucoside on cotton pellet-induced granuloma in rats.

Treatment	Weight of Granuloma (mg)	Inhibition (%)
Control	27.02 ± 1.92	--
Test Compound (5 mg/kg)	20.78 ± 1.23 *	23
Test Compound (10 mg/kg)	18.13 ± 0.98 *	33
Test Compound (15 mg/kg)	15.83 ± 1.02 *	41
Indomethacin (10 mg/kg)	14.55 ± 1.35 *	46

The results are represented by mean ± SEM (n = 6). * *p* < 0.05 compared to control group.

## Supplementary Materials

The following are available online at https://www.mdpi.com/2305-6320/6/2/59/s1, Figure S1: IR spectra of the test compound, Figure S2: Mass spectra of the test compound, Figure S3: ^1^H-NMR spectra of the isolated compound.
