# Supplementary material for: Analgesic and Anti-Inflammatory Activities of Quercetin-3-methoxy-4′-glucosyl-7-glucoside Isolated from Indian Medicinal Plant Melothria heterophylla
Source: Medicines (Basel). 2019 May 27;6(2):59. doi: 10.3390/medicines6020059 (PMC6631596; doi:10.3390/medicines6020059)
Supplement: Supplementary file 1 [file medicines-06-00059-s001.pdf]

Supplementary Materials: Analgesic and Anti-Inflammatory Activities of Quercetin-3-methoxy-4′-glucosyl-7-glucoside Isolated from Indian Medicinal Plant *Melothria heterophylla*

Arijit Mondala, Tapan Kumar Maityb and Anupam Bishayee

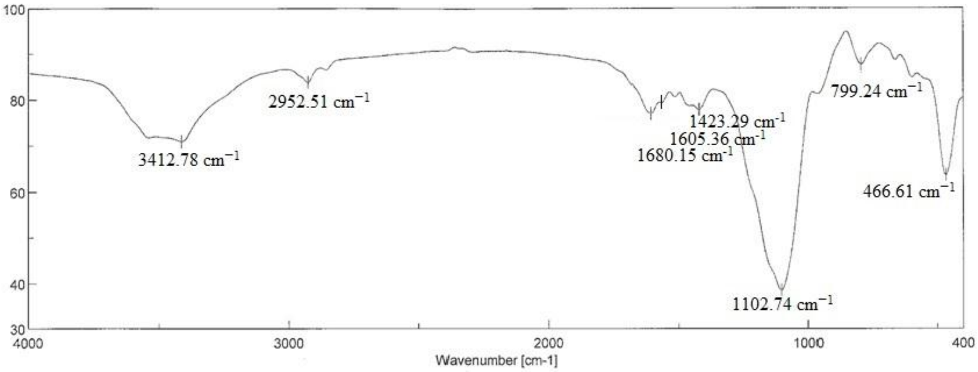

Figure S1. IR spectra of the test compound.

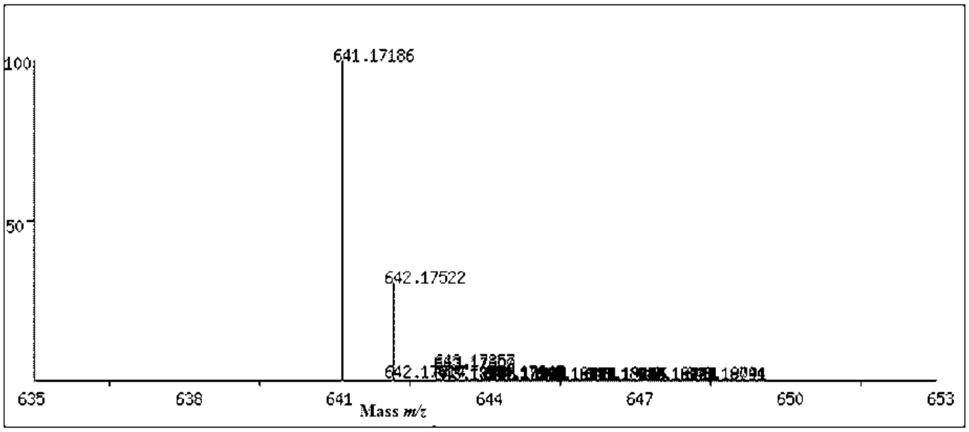

Figure S2. Mass spectra of the test compound

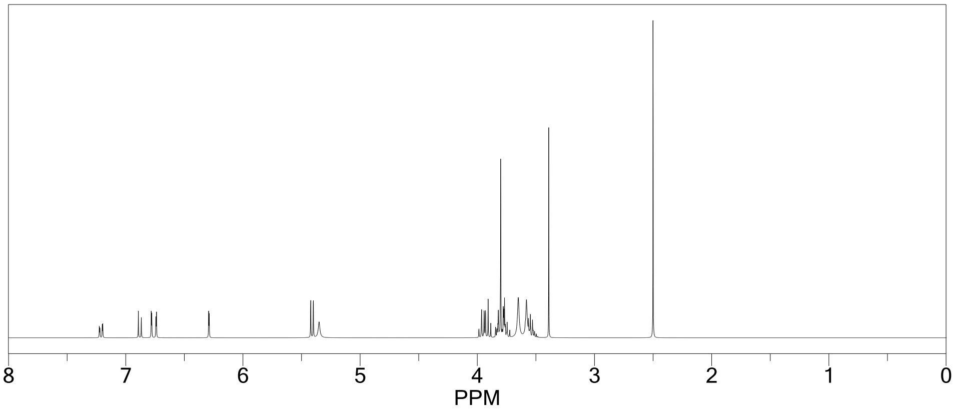

Figure S3. ^{1}H-NMR spectra of the isolated compound.
